# Mr. Fogo on Twin Cases

**Published:** 1811-08

**Authors:** A. Fogo

**Affiliations:** Newcastle


### To the Editors of the Medical and Physical Journal.

GENTLEMEN,

IN your last No. 148, p. 185, I observed a paper on the case published by Jones, on the subject of twins, by one who calls himself an Essex Practitioner, and very modestly, an inexperienced practitioner. The writer "condemned himself for rashness," for doing, what in my humble opinion, founded on both theory and practice, he ought to have done two hours before. He says, "in the absence of any particular bad symptom, delay cannot be very hazardous, whereas, producing delivery '*per artem*,' is always attended with danger, both to mother and child." In the above, he may, possibly, be deceived, from not *seeing* any particular bad symptom; but he little knew or suspected what was going on in the dark: and the hidden works of darkness ought always to be suspected. Was he certain that no unseen hæmorrhage was going on. Was he certain that the placenta of the first child continued to adhere firmly to the uterus? Was he certain that the vessels of the two placentas did not anastomose, and the first being detached, hæmorrhage would take place from both uterus and placenta. He says "no hæmorrhage succeeded the birth of the child, the placenta was retained by the undelivered child." So was any blood which had escaped from the vessels of the uterus or placenta; and as there was no appearance of blood there was no alarm. "I waited two hours, no pains returned, nor was there any hæmorrhage, but a great degree of languor." The cause of the last will soon appear. During these two hours the woman was losing blood; but being prevented passing the os externum, by the placenta of the first, and by the body, membranes, and waters of the second child, it did not, or could not, make its appearance. As soon as he delivered the second child, "flooding to an alarming extent instantly succeeded; and after bringing away the two placentas, the gush was such as I had never before witnessed." These, the flooding and the gush, were composed of the great pool of blood, which, I take for granted, was accumulating during two hours, from the detachment of the first placenta, which was confined, as mentioned above, till a passage was opened for it. The loss of this blood very readily accounts for the "great degree of languor." The writer's expression helps to confirm me in the above opinion. He says "the flooding soon abated, but this arose from the sunk state of the

Q 2 patient, patient, who had, in a few moments, lost not less than six or seven pounds of blood." The loss of so much blood in a few moments, in my opinion, proves that it was, as hinted above, ready, in an extravasated state, to gush out at the first opportunity. I deny that the vessels of any uterus can discharge six or seven pounds of blood in a few moments: is he aware that moments signify the more usual modern name of seconds?

The writer then condemns himself for what he did, forms a resolution, whenever he meets with twins in future, to deliver the first '*per artem*,' and leave the second to nature. However he may despise my advice, which is just the reverse, I caution him, before he unsheaths his *Tire-tetes*, that he is perfectly certain that there are two or three children. If he ever attended any respectable teacher, or read any respectable book on the subject, he must have heard or seen that few or no practitioners have been able to discover a twin case before the first is born. If he can point out any certain mode of discovering twins or triplets, erit vetustissimis nobis obstetricibus magnus Apollo. I may further presume to add, that if he had been of his present opinion and left the second to nature other two hours, he would, very probably, have witnessed the death of both mother and child.

He has contradicted the assertion he set out with, "that delivery *per artem* is always attended with danger to the mother and her offspring." Instead of which he had recourse to delivery *per artem*, and although, in my opinion, too long delayed, yet it narrowly saved the mother; there is no account of the child.

Dr. Denman, in a late publication, says, the science of midwifery has been improved in the present age beyond our utmost expectations; or words to that purpose, as I have not the book before me. I confess I am of a very different opinion, and have not kept these opinions secret; particularly in the case treated above. I am inclined to believe that the doctrines the philosophising and forbearing Dr. Hunter has instilled into his pupils will continue to prevail, and that the science is rather in a retrograde state. Do nothing; give no assistance; leave all to nature, but *guard the perineum.* Some observations on this fashionable forbearing practice will, possibly, be made in future. And also on the absurdity of the practice of guarding the perineum, opposing the passage of the child, and protracting the labour, which at the best is bad and long enough.

I am, &c.

A. FOGO.

*Newcastle, June 8th,* 1811.

